# Out with the old: Hsp90 finds amino acid residue more useful than co-chaperone protein

**DOI:** 10.15698/mic2017.08.586

**Published:** 2017-08-01

**Authors:** Abbey D. Zuehlke, Leonard Neckers

**Affiliations:** 1 Urologic Oncology Branch, Center for Cancer Research, National Cancer Institute, 9000 Rockville Pike, Bethesda, MD 20892, USA.

**Keywords:** co-chaperone, posttranslational modification, ATPase cycle, client protein

## Abstract

Redundant functions maintained from single to multi-cellular organisms have made *Saccharomyces cerevisiae* an important model for the analysis of conserved com-plex cellular processes. Yeast has been especially useful in understanding the regulation and function of the essential molecular chaperone, Heat Shock Protein 90 (Hsp90). Research focused on Hsp90 has determined that it is highly regulated by both co-chaperones and posttranslational modifications. A recent study per-formed by (Zuehlke *et al.*, 2017) demonstrates that the function of one co-chaperone in yeast is replaced by posttranslational modification (PTM) of a single amino acid within Hsp90 in higher eukaryotes.

As an important regulator of cellular pathways, Hsp90 functions include assisting the proper folding and activation of approximately 10% of cellular proteins, termed clients. Hsp90 exists as a dimer with each protomer containing an N-terminal ATP-binding motif, a middle domain, and a C-terminal dimerization domain. Given its complex functions in the cell, Hsp90 relies on several forms of regulation including nucleotide interaction, co-chaperone assistance, and posttranslational modification. Overlapping regulatory pathways and in vivo functions make yeast Hsp90 a useful model for studying human Hsp90. Yeast and human Hsp90 share 69% sequence identity and of the 15 co-chaperones natively found in yeast, 14 are conserved in higher eukaryotes. We found that the one co-chaperone lost during eukaryotic evolution to multi-cellularity, Hch1, may have been replaced by the acquisition of a kinase capable of phosphorylating tyrosine 627 in Hsp90.

Hch1 is a co-chaperone first thought to be a weak modulator of Hsp90’s ATPase domain. Due to the presence of the stronger ATPase regulatory co-chaperone Aha1, it was predicted that Hch1 function was unnecessary, resulting in its evolutionary loss. Recent work, however, identified functions unique to Hch1. The presence of Hch1 was found to sensitize yeast to Hsp90 mutation and pharmacological inhibition. Our work also demonstrates that Hch1 exacerbates the client chaperoning and growth defects of the Hsp82-W585T mutant, revealing a more important role for Hch1 in Hsp90 function and leaving us to wonder how its tasks are replaced in multi-cellular eukaryotes.

The Y627 residue located on human Hsp90 was previously determined to be phosphorylated by Yes kinase. Phosphorylation of this site was found to reduce Hsp90-client interactions. This site in yeast, although also a tyrosine, is not phosphorylated. As this amino acid is positioned in a similar region as the W585T mutation, and its phosphorylation impacts client chaperoning, we hypothesized that posttranslational modification of this site may phenocopy the role played by Hch1. In agreement with our hypothesis, evolutionary appearance of Yes kinase and loss of *HCH1* coincides with acquisition of multicellularity.

We used genetic mutation to determine if phosphorylation of Y627 has a similar impact on Hsp90 function as does the presence of Hch1. Phosphomimetic and non-phosphorylatable mutants were introduced into the yeast Hsp90 protein, Hsp82. Client activity assays and growth experiments demonstrated that the phosphomimetic, but not non-phosphorylatable, mutant has a similar effect on Hsp90 function as does the presence of Hch1.

In order for Hsp90 to properly chaperone its client proteins, it proceeds through several nucleotide-influenced conformations. Mutations that prevent Hsp90 from proceeding into the closed (N-domain dimerized) conformation have a dramatic effect on client chaperoning and cell growth. Because Hch1 and Y627 phosphorylation have a similar negative impact on Hsp90 function, we postulated that they may similarly prevent Hsp90 from proceeding into the closed state. Supporting this hypothesis, a mutation stabilizing the closed conformation of Hsp90 was able to rescue the client and growth defects associated with either overexpression of *HCH1* or phosphorylation of Y627. ATP-stabilized conformations acquired by Hsp90 also differentially affect co-chaperone interactions. Binding of the non-hydrolyzable form of ATP, AMP-PNP, to Hsp90 stabilizes the closed conformation. This specific Hsp90 configuration results in increased interaction with the co-chaperone Sba1. Therefore, Sba1 interaction in the presence of AMP-PNP can be used to determine whether or not the stable closed state of Hsp90 can be readily achieved. Our data demonstrate that both* HCH1 *and Y627 phosphorylation reduce the ability of Hsp90 to attain the closed conformation, providing an explanation for their similar impact on client chaperoning and cell growth (Figure 1).

**Figure 1:**
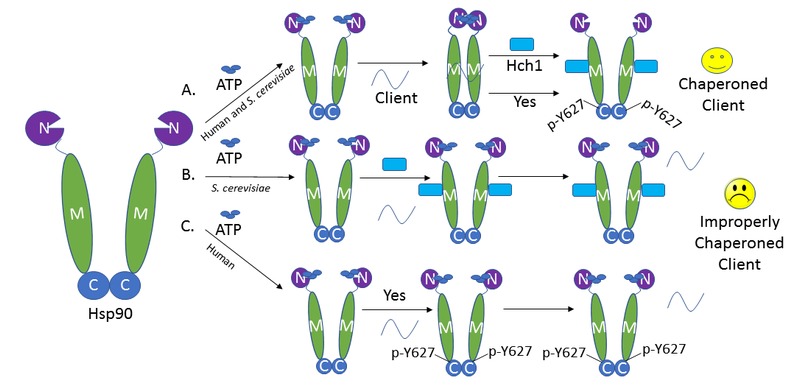
FIGURE 1: Hch1 interaction to yeast Hsp90 and phosphorylation of Y627 in human Hsp90 impact client chaperoning through restricting Hsp90 conformational changes. **(A)** Normal ATP-driven client chaperoning in both humans and yeast. Appropriate interaction of Hsp90 with Hch1 (yeast) or phosphorylation of Hsp90-Y627 (human) promotes release of chaperoned client. **(B)** Deregulated Hch1 interaction with yeast Hsp90 restricts its conformational flexibility, resulting in reduced client chaperoning. **(C)** Deregulated phosphorylation of human Hsp90 at Y627 phenocopies consequences of deregulated Hch1 interaction in yeast.

Given the many functional similarities between Hch1 and Hsp90-Y627 phosphorylation, we postulated that the simultaneous presence of both the co-chaperone and this Hsp90 PTM would represent a significant burden to the cell. Confirming this hypothesis, we found that yeast co-expressing Hch1 and the phosphomimetic Hsp90-Y627 mutant were temperature sensitive. Taken together with the fact that acquisition of Yes kinase and loss of* HCH1* occurred at approximately the same point in eukaryotic evolution, our data support the concept of evolutionary replacement of Hch1 in yeast by reversible phosphorylation of Hsp90-Y627 in higher eukaryotes.

